# Education and training to support physiotherapists working in dementia care: a scoping review protocol

**DOI:** 10.12688/hrbopenres.13219.2

**Published:** 2021-06-22

**Authors:** Trish O'Sullivan, Tony Foley, Joseph G. McVeigh, Suzanne Timmons

**Affiliations:** 1 Discipline of Physiotherapy, School of Clinical Therapies, University College Cork, Cork, Ireland; 2 Department of General Practice, School of Medicine, University College Cork, Cork, Ireland; 3 Centre for Gerontology and Rehabilitation, School of Medicine, University College Cork and Mercy University Hospital, Cork, Ireland

**Keywords:** Dementia, Physiotherapy, Education, Training

## Abstract

**Background: **The care of people with dementia is of global concern. Physiotherapeutic intervention can be of benefit to patients with dementia. Physiotherapists can play a role in assessment, falls prevention, pain management and gait re-education. Dementia care forms a significant part of the workload of a physiotherapist. However, there is a paucity of evidence on what constitutes effective education and training for physiotherapists working in dementia care.

**Objective: **This scoping review aims to explore and map the evidence on education and training for physiotherapists working in dementia care.

**Inclusion criteria: **Studies that explore dementia training and/or education for physiotherapists or for multidisciplinary teams, in which physiotherapists have been included. Studies that explore student physiotherapy training will also be considered. Qualitative, quantitative, mixed methods studies, case studies and observational studies will be included.

**Methods:** This scoping review will follow the Joanna Briggs Institute (JBI) methodology for scoping reviews. Databases to be searched as part of this review include: Medline, SocINDEX, CINAHL and, PsycINFO, with no limitation on publication date. Google Scholar and Open-Grey will be searched for grey literature, limited to the first 100 searches. Titles and abstracts will be screened for inclusion and identified full texts reviewed independently by two reviewers. Data will be extracted using a draft data extraction tool based on the JBI data extraction tool. A chronological narrative synthesis of the data will outline how the results relate to the aims and objective of this scoping review.

## Introduction

Dementia is one of the greatest health and social care challenges of our time ^1^. Dementia is characterised by progressive cognitive impairment in domains such as memory, orientation, comprehension, language and judgement ^2^. The complexity of dementia, coupled with the significant health and social care costs make dementia a major challenge to our healthcare system. As Ireland’s ageing population continues to grow, it is estimated that by 2036, there will be over 112,000 adults in Ireland living with dementia ^3^. The consequences of dementia for the individual and family are profound as it is associated with impairment in both physical and functional ability, as well as non-cognitive symptoms (often called behavioural and psychological symptoms of dementia). Physical impairments associated with dementia include impaired mobility, reduced muscle strength and poor balance while non-cognitive symptoms include agitation, anxiety, depression and delusions ^4^. Rehabilitation interventions are an important component of the management of those with dementia and physiotherapy plays a key role in many aspects of dementia care, including the maintenance of mobility, falls risk assessment and strength training ^5^. Even though physiotherapists working in acute and primary care settings carry a significant caseload of patients with dementia ^6^, very little formal undergraduate and post-graduate training is available to allied health care professionals ^7^. The World Health Organisation has acknowledged that developing the knowledge and skills of all healthcare professionals who are involved in dementia care is a priority ^8^. Indeed the Irish National Dementia Strategy has included training and upskilling of healthcare professionals in dementia care as a primary objective ^3^.

Understanding what constitutes effective education, for those caring for patients with dementia in the healthcare setting is an ongoing challenge ^9^. The diversity of knowledge, abilities, skills and qualities required to be a competent healthcare professional in dementia care highlights both the complexity and importance of education in this area. However, there remains a gap in the literature on what effective dementia care training for physiotherapists should address, consist of, how it should be delivered and how it should be evaluated. Even though basic didactic knowledge is outlined in the curricula of most allied health professional courses ^10^, Surr and Gates argued that the ability to effectively transfer theoretical knowledge from the curricula to clinical practice remains challenging ^9^. A preliminary search of the JBI Database of Systematic Reviews and the Cochrane Library, Medline and CINAHL databases did not find any scoping reviews of dementia educational interventions for physiotherapists. Similarly, a search of the PROSPERO database found no similar systematic review protocol registered or currently ongoing. However, previous research ^9, 11, 12^ has explored what constitutes effective dementia training and education for the wider health and social care workforce. For instance, a critical synthesis ^9^ that analysed dementia training for hospital staff examined staff’s knowledge gains, changes in attitudes, confidence and behaviour change. This review found that the aspects of dementia training that were most effective are those strategies that participants can apply in their day to day practice and are related to the content and delivery ^9^. The 20 papers included in this review by Surr and Gates ^9^, evaluated 16 different training programmes, however, the methods of evaluation varied from study to study. Moreover, it is difficult to quantify perceived increase in knowledge as a result of training, as many questionnaires measuring knowledge are non-validated ^9^. It is evident that dementia training can lead to more positive attitudes which in-turn results in improved patient outcomes ^13^. Yet, in spite of this, there remains a paucity of evidence in the literature as to what constitutes effective dementia training for physiotherapists and more importantly, what type of curriculum will translate into better patient care.

### Aim

This scoping review aims to explore and chart the evidence relating to education and training for physiotherapists working with people with dementia with a view to identifying any gaps within the literature.

## Objectives

- To identify studies that have evaluated physiotherapy dementia educational interventions.
- To critically appraise included studies, in order to update the current evidence base.
- To use the findings of the review to inform the design and delivery of a dementia educational programme for physiotherapists.

### Purpose

A greater understanding of what constitutes effective dementia education and training for physiotherapists will lead to appropriately designed educational interventions for physiotherapists, ultimately leading to the opportunity for enhanced patient care.

## Inclusion criteria

### Population

The review will consider studies that include dementia education or training for both qualified physiotherapists and student physiotherapists. It will also include studies that looked at multi-disciplinary dementia training only if physiotherapy was an included profession.

### Concept

The proposed scoping review is designed to explore education and training for physiotherapists working in dementia care. Therefore all studies with a focus on any aspect of physiotherapy education and training will be considered. Education is a learning process that deals with unknown outcomes, and circumstances which require complex knowledge synthesis, skills and experience to solve problems ^14^. Training has application when there is some recognised skill that has to be mastered and practice is required for the mastery of it ^14^. Effective learning in health care education includes elements of training set in the context of life- long learning. As dementia care incorporates clinical, social, ethical and medical issues, it is important that concepts from both education and training are included.

As defined by the World Health Organisation, knowledge translation is “the synthesis, exchange, and application of knowledge by relevant stakeholders to accelerate the benefits of global and local innovation in strengthening health systems and improving people’s health” ^15^. Knowledge translation needs to move beyond the simple dissemination of knowledge to the actual use of knowledge. Within the Cochrane Collaboration, the Cochrane Effective Practice and Organisation of Care Group (EPOC) review the effects of knowledge translation activities ^16^ and have explored the effectiveness of professional behaviour change strategies. The EPOC give clear definitions of education meetings, education outreach and audit and feedback ^16^. The authors will be guided by the EPOC to ensure the inclusion of studies that meet the definition of these educational interventions. 

### Context

This scoping review aims to establish the breadth and extent of the current literature published on dementia training for physiotherapists and student physiotherapists. Therefore, studies conducted in any setting (acute/primary care/residential care) or any educational setting in any geographical location will be considered. The WHO describes primary care as “first contact, accessible, continued, comprehensive and co-ordinated care” ^17^.

### Types of studies

This scoping review will look at all, qualitative, quantitative and mixed methods studies that explore dementia education and physiotherapy. Case studies, as well as observational studies will also be included.

## Methods

This scoping review will follow the Joanna Briggs Institute (JBI) methodology for scoping reviews ^18^. This protocol was registered with Open Science Framework on 26 October 2020.

### Search strategy

As recommended in the JBI guidelines, a three step search strategy will be used ^18^. The first step, which has already been completed, involved a broad search of Medline (via EBSCO) and CINAHL using keywords for physiotherapy, dementia and education. This initial search was then followed by an analysis of the text words found in the identified titles and abstracts ^19, 20^. This ensured that relevant literature was captured. The search strategy was developed by one reviewer (TOS) with assistance from a librarian in University College Cork. The final search strategy for CINAHL is presented in Table 1. The final step in the search strategy will include a detailed search of the reference lists of identified studies. Databases to be searched as part of this review include: Medline, CINAHL, PsycINFO and SocINDEX. Google Scholar and Open-Grey will be searched for grey literature. Only studies published in English will be considered.

**Table 1.** Search Strategy: CINAHL plus full text. Date of Search: 19/09/20.

Number	Search Terms	Records Retrieved
#1	Physiotherapy or Physical Therapy or Physiotherapist or Rehabilitation	124,447
#2	Dementia or Alzheimer’s Disease or Cognitive Impairment or Dementia Vascular or Memory Loss	91,389
#3	Education or training or learning or teaching	420,523
#4	#1 and #2	2,589
#5	#4 and #3	399

### Study selection

Following the search, all identified citations will be collated and uploaded to EndNote X9.2 and duplicates removed. Titles and abstracts will then be reviewed independently by two reviewers (TOS and TF) for assessment against inclusion criteria. Where uncertainty occurs, a third reviewer (JMcV) will be consulted. The full texts of selected studies will be then screened for inclusion. Full text studies that do not meet the inclusion criteria will be excluded and reasons for exclusion recorded. The results of this search will be comprehensively detailed and reported in a Preferred Reporting Items for Systematic Reviews and Meta-Analysis (PRISMA) flow diagram ^21, 22^.

### Data extraction

Data extraction will focus on identifying and charting data relating to physiotherapy education in dementia care. Data will be extracted using a draft data extraction tool based on the JBI data extraction tool ^18^. The data extracted will include study characteristics such as author, year, type of study, publication title, country, clinical setting, participants, purpose, education content, mode of delivery, key findings, barriers and facilitators to learning and limitations. The Kirkpatrick Framework ^23^, a hierarchy of evaluation of training, will be used to classify data extracted. The Kirkpatrick Framework ^23^ was chosen as it is widely used to evaluate educational interventions in healthcare. The four level model outlined by Kirkpatrick comprises of 1) reaction, 2) learning, 3) behaviour and 4) results ^23^. The draft data extraction tool will be independently piloted on three papers by two reviewers (TOS, JMcV) and modified where necessary. Modifications will be detailed in the full scoping review report. Data will be extracted by one reviewer (TOS) and reviewed by another reviewer. Any disagreements that arise will be resolved through discussion or with a third reviewer. Missing or additional data will be obtained by contacting study authors where required.

### Data presentation

A chronological narrative synthesis of the data will outline how the results relate to the aims and objective of this scoping review. The review will characterise what constitutes effective dementia care training for physiotherapists, the various teaching and learning approaches used and the outcomes of the training intervention. Kirkpatrick’s framework will be used to report the effectiveness of the educational intervention. This will involve looking at reaction and satisfaction of participants, learning and knowledge, participants behaviour and patient outcomes. The various dementia training interventions will be tabulated in suitable categories.

### Dissemination of information

The findings of this review will be disseminated in several ways. The scoping review will be published in an international peer reviewed journal, the results will also be presented at national and international conferences. More locally, the findings of the review will be disseminated through the Dementia Research Network Ireland (DRNI) and to clinical colleagues in the Health Service Executive (HSE). 

### Study status

This study is at stage 1: scoping review to explore and chart the evidence relating to education and training of physiotherapists working in dementia care.

## Data availability

### Underlying data

No data are associated with this article.
