# Supplementary material for: Education and training to support physiotherapists working in dementia care: a scoping review protocol
Source: HRB Open Res. 2021 Jun 22;4:27. Originally published 2021 Mar 10. [Version 2] doi: 10.12688/hrbopenres.13219.2 (PMC8167503; doi:10.12688/hrbopenres.13219.2)
Supplement: Peer review history

# Peer review: Reviewer response for version 2

**DOI:** 10.21956/hrbopenres.14518.r29717

**Published:** 2021-06-22

**Authors:** Amanda Phelan

**Affiliations:** 1 School of Nursing & Midwifery, Trinity College Dublin, Dublin, Ireland

I am happy with this version apart from some minor punctuation errors.

Is the study design appropriate for the research question?

Yes

Is the rationale for, and objectives of, the study clearly described?

Yes

Are sufficient details of the methods provided to allow replication by others?

Yes

Are the datasets clearly presented in a useable and accessible format?

Yes

Reviewer Expertise:

Safeguarding, public health, older people, missed care

I confirm that I have read this submission and believe that I have an appropriate level of expertise to confirm that it is of an acceptable scientific standard.

# Peer review: Reviewer response for version 1

**DOI:** 10.21956/hrbopenres.14373.r29352

**Published:** 2021-05-28

**Authors:** Amanda Phelan

**Affiliations:** 1 School of Nursing & Midwifery, Trinity College Dublin, Dublin, Ireland

Thank you for the opportunity to review this paper. The focus of enhancing skills in professionals' support of people living with dementia is a key priority, particularly in the context of an increasing ageing population.

 Physiotherapists have a key role in this regard, yet having bespoke education and training may be limited, despite the high potential for enhancement of quality of life.

 From a cursory review of this area within the context of a literature search, there seems to be scant literature on physiotherapy and education and training. The literature appears to generally describe contributions (actual and potential) of physiotherapy and treatment efficacy. Consequently, the focus to widen the search to include multi-disciplinary teams within which physiotherapists deliver care allows more scope, but care will have to be taken to isolate the education and training needs for physiotherapy professionals in particular. 

 Defining what types/forms of education and training would be useful: within undergraduate programmes for physiotherapists, within postgraduate for physiotherapists, or within interdisciplinary graduate programmes.

 In addition, identifying what is 'training'-is this bespoke to the discipline, inter-discipline, accredited continuous professional education programmes, study days etc. Is there a focus on competencies acquisition in clinical practice (and validated by a mentor?).

 The process of the literature search is enhanced by the skills of the subject librarian and the mapping to Kirkpatrick's framework will enable a credible anchor for classification.

 The methods for data extraction and resolving any disputes are transparent. Details of data dissemination are clear and suggest this is also of interest to physiotherapists outside the HSE (ie private hospitals) and has the potential to raise areas of competency development potential of private physiotherapists who deliver care in nursing homes.

Is the study design appropriate for the research question?

Yes

Is the rationale for, and objectives of, the study clearly described?

Yes

Are sufficient details of the methods provided to allow replication by others?

Yes

Are the datasets clearly presented in a useable and accessible format?

Yes

Reviewer Expertise:

safeguarding, public health, older people, missed care

I confirm that I have read this submission and believe that I have an appropriate level of expertise to confirm that it is of an acceptable scientific standard.

# Peer review: Reviewer response for version 1

**DOI:** 10.21956/hrbopenres.14373.r29386

**Published:** 2021-05-28

**Authors:** Louise Hopper

**Affiliations:** 1 School of Psychology, Dublin City University, Dublin, Ireland

This study comprises of the protocol for a scoping review of dementia care training for physiotherapists. A clear rationale is provided for the need for this review. Although previous reviews have been conducted relating to dementia care needs and dementia care training in healthcare professionals more broadly, no review to date has focused solely on physiotherapists despite previous research indicating that the training that produces the best outcome is that which identifies care elements that can be successfully implemented in daily interactions. The protocol methodology provides sufficient information to replicate the study. A validated model (JBI) will be followed, which is a strength of this protocol.

Is the study design appropriate for the research question?

Yes

Is the rationale for, and objectives of, the study clearly described?

Yes

Are sufficient details of the methods provided to allow replication by others?

Yes

Are the datasets clearly presented in a useable and accessible format?

Not applicable

Reviewer Expertise:

Dementia care, policy, education and psychosocial interventions

I confirm that I have read this submission and believe that I have an appropriate level of expertise to confirm that it is of an acceptable scientific standard.

# Peer review: Reviewer response for version 1

**DOI:** 10.21956/hrbopenres.14373.r29074

**Published:** 2021-03-30

**Authors:** Katherine Lawler

**Affiliations:** 1 Wicking Dementia Research and Education Centre, University of Tasmania, Hobart, Tas, Australia

Thank you for the opportunity to review this paper. I wish to thank the authors for their commitment to pursuing research which ultimately aims to improve health and wellbeing outcomes for people living with dementia.

 This paper describes a protocol for a scoping review about education and training for physiotherapists both practicing and at undergraduate level, in the field of dementia care. The authors present a compelling argument for the importance of dementia, the role physiotherapists can play, and the global imperative to improve education and training for health professionals working in this field.

 I have included a few brief comments below for consideration. However, the paper in its current form provides an excellent platform for building a scoping review upon, particularly with the authors' careful use of JBI methodology and the PRISMA-ScR checklist. I look forward to reading the final results.

***Introduction***

- **Comment 1: **I hesitated around the description of the gap in the literature. At first glance I felt concerned that the review would yield no results, as the gap was discussed quite extensively. On second reading I wondered if the gap related more to a gap in synthesised evidence. Perhaps this could be clarified. If there is a paucity of evidence available regarding dementia training for physiotherapists, does the scope of the review need widening? Perhaps including other allied health professions, for example?

***Inclusion Criteria***

- **Comment 2:** World Health Organisation (rather than Healthcare)
- **Comment 3:** Consider whether defining  'training' vs 'education' is relevant for this review. Both are in the title but there appears to be a stronger emphasis on training. I would suggest including both is valuable but defining terms may help.

***Methods***

- **Comment 4:** ERIC may be a good database to consider, with an education focus (but I will defer to your research librarian!)
- **Comment 5: **It could also be interesting to map specific gaps in physio education/training relating to Kirkpatrick's framework, to inform future research.

Is the study design appropriate for the research question?

Yes

Is the rationale for, and objectives of, the study clearly described?

Yes

Are sufficient details of the methods provided to allow replication by others?

Yes

Are the datasets clearly presented in a useable and accessible format?

Not applicable

Reviewer Expertise:

physiotherapy; dementia care; systematic reviews

I confirm that I have read this submission and believe that I have an appropriate level of expertise to confirm that it is of an acceptable scientific standard.
